# Combining polyesters of citric and azelaic acids to obtain potential topical application biomaterials with antimicrobial activity

**DOI:** 10.3389/fbioe.2025.1579630

**Published:** 2025-06-12

**Authors:** Aleksandra Bandzerewicz, Anna Herman, Ewa Dutkowska, Klara Niebuda, Paweł Ruśkowski, Agnieszka Gadomska-Gajadhur

**Affiliations:** Faculty of Chemistry, Warsaw University of Technology, Warsaw, Poland

**Keywords:** polycitrate, polyazelate, polymer films, wound dressings, *Pseudomonas aeruginosa*, antimicrobial

## Abstract

Biomaterials with antimicrobial properties are a key research area due to the increasing threat of infections and the growing resistance of microorganisms to existing antibiotics. The aim of the study was to produce thermally cross-linked polymer films based on poly(1,5-pentanediol azelate) and poly(1,4-butanediol citrate) with antimicrobial activity for medical applications. Well-formed, cross-linked, flexible materials differing in appearance depending on the conditions of the cross-linking process were obtained. In general, a lower cross-linking temperature was found to promote less brittle and more flexible films with greater structure uniformity. The polymer films had hydrophilic surfaces (water contact angle 40°–60°). All polymer films maintained integrity after immersion in PBS buffer. Most likely, the lower hydrophilicity of the polyazelate phase limited their degradation. A modified time-kill procedure (ASTM E2315-23) was performed to test the antimicrobial properties of the films against *Pseudomonas aeruginosa*, *Staphylococcus aureus*, and *Candida albicans*. The antimicrobial activity of polycitrate-based films against *P*. *aeruginosa* has been reported with >90% reduction of the pathogen after 6 h of contact and 100% biocidal effect after 24 h. The antimicrobial activity of the film is pH-based. The biocidal effect of polycitrate film against *P. aeruginosa* is the most important and promising result, especially given the resistance of the pathogen to commonly used antibiotics.

## 1 Introduction

Over the past century, the excessive and inadequate use of antibiotics has led to the natural selection of drug-resistant bacteria. Aside from the increased severity and prolonged and more complex treatment required, microbial infections also become significantly more expensive and difficult to diagnose and overcome. What initially was mainly associated with the gatherings of critically ill and immunosuppressed patients has now extended beyond the hospital setting (Alanis, 2005; Cabello, 2006; Bell et al., 2014; Dixon and Duncan, 2014).

Antibiotic resistance is usually considered concerning orally administered drugs. However, it is not the sole issue of this kind. Antibiotic resistance is of increasing concern in topical infections, such as acne treatment, specifically in terms of the long-term use of antibiotics suppressing *Propionibacterium acnes* (Patel et al., 2010; Andriessen and Lynde, 2014; Walsh et al., 2016). In general, every time a naturally balanced skin barrier becomes compromised due to the development of a wound, the possibility of microbial penetration, wound colonization, and infection increases rapidly. The isolation of antibiotic-resistant microorganisms, which is enhanced by the widespread use of antibiotics, includes wound management as well (Wright et al., 1998). Particularly crucial challenges are set by *Staphylococcus aureus* and *Pseudomonas aeruginosa*, which are the most common bacteria isolated from chronic wounds. These pathogens are capable of forming a strong biofilm, subsequently impairing the healing process and leading to the development of antibiotic resistance (Serra et al., 2015). The resistance of *P. aeruginosa* to commonly used antibiotics is alarming as it constantly grows (Bashir Ahmed, 2016; Dou et al., 2017; Farooq et al., 2019).

**FIGURE 1:**
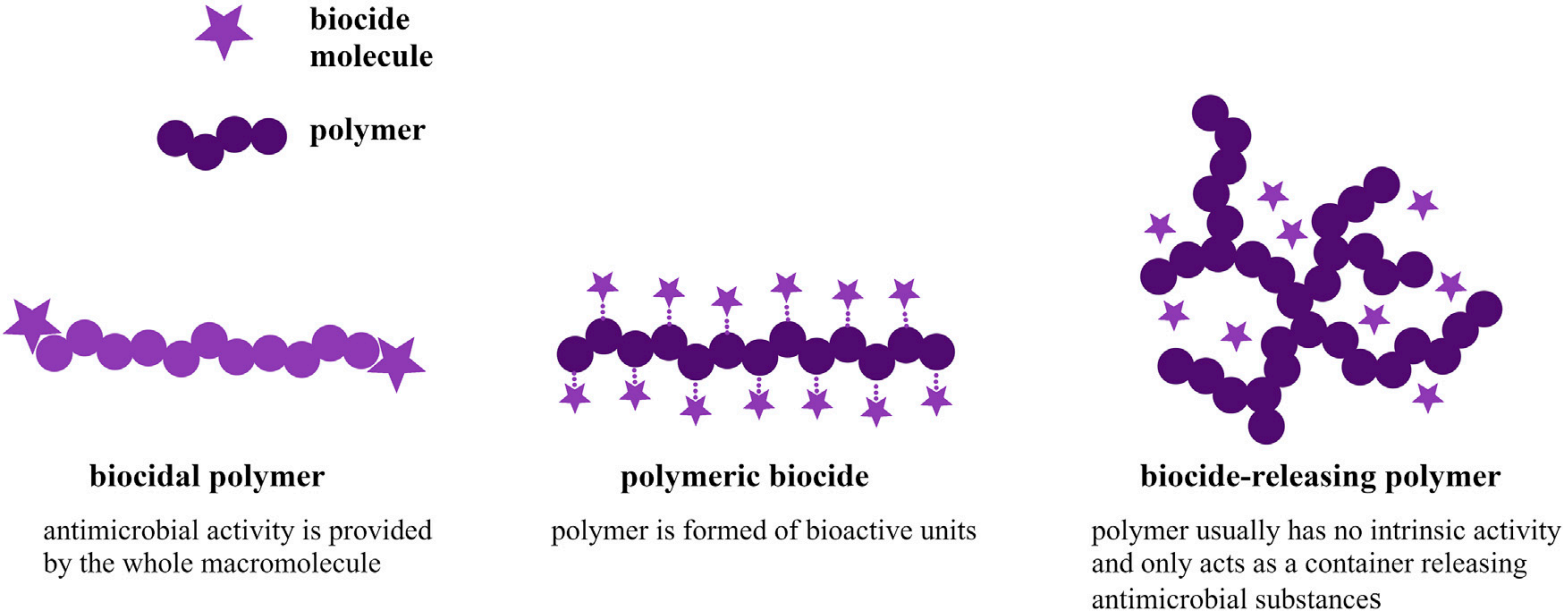
Types of antimicrobial polymers based on the type of the polymeric system.

When microorganisms adhere to the surface, they excrete a mixture of polysaccharides, proteins, and other polymers to form a so-called biofilm, become embedded within, and enable themselves to anchor to the substrate. This is a fundamental process because if a defective biofilm is formed, it becomes impossible for microorganisms to grow properly (Huang et al., 2016). Biofilms are difficult to remove and resistant to many biocides. For this reason, the design of various antimicrobial protection products and devices is based on the process of inhibiting biofilm formation and microbial attachment (Mahanta et al., 2021; Palza et al., 2022). Given the declining ability of antibiotics to effectively combat microbes, polymeric materials with unique antimicrobial properties are of increasing interest (Hippius et al., 2014; Yu et al., 2014; Zhou et al., 2014; 2015; Liu et al., 2015; Shahzad et al., 2015; Zhang et al., 2015). Depending on the type of the system, polymers with antimicrobial activity can be divided into three groups Figure 1: (1) biocidal polymers, which exhibit intrinsic antimicrobial activity; (2) polymeric biocides, whose mechanism for combating microorganisms is based on polymeric skeletons with attached biocide molecules; and (3) polymers loaded with biocide molecules that are released into the microenvironment (Santos et al., 2016).

Polymeric materials can be divided into passive and active types based on the mechanism of their behavior towards microorganisms. The passive material does not actively kill microorganisms but only reduces protein adsorption on the substrate and prevents pathogen adhesion. One of the most commonly used passive antimicrobial polymers is poly(ethylene glycol) (PEG) (Huang et al., 2016; Barnes et al., 2019). Active-type mechanism of killing microorganisms involves several ways of action, including (i) interaction with and disruption of negatively charged microbial cell membranes, (ii) penetration into the microbe and inhibition of nucleic acid and protein synthesis, (iii) chelation of metal ions and paralysis of nutrient and oxygen supply, (iv) inhibition of vital functions by free radical attack (Roy and Gupta, 2003; Bugnicourt et al., 2014; Jain et al., 2014; Qiu et al., 2020; Babutan et al., 2021; Roy et al., 2022; Liu et al., 2024).

As regards biodegradable polyesters, it is oftentimes considered that the reason for their antimicrobial activity lies mostly in the acidic by-products of their hydrolytic degradation, the concentration of which may result in a local pH drop. Since the growth of most common pathogens is ineffective or even completely inhibited if exposed to suboptimal pH (usually outside of the four to nine window), organic acids and their polyester derivatives have proven to be of use as antimicrobial agents. Some of these applications include combining polylactide, poly(lactide-co-glycolide), poly-ε-lysine and chitosan for their antimicrobial activity against *Escherichia coli*, *Staphylococcus epidermidis*, *S. aureus*, *B. subtilis*, or *P. acnes* (Korting et al., 1992; Presser et al., 1997; Klein et al., 2009; Hild et al., 2013; Jain et al., 2014; Gritsch et al., 2018).

Both azelaic and citric acid have been studied for their bactericidal effect against multiple Gram-negative and Gram-positive microorganisms (Holland and Bojar, 1993; Georgopoulou et al., 1994; Charnock et al., 2004; Sieber and Hegel, 2013; Eliuz, 2020). Polymer films made of citric acid polyesters were shown to exhibit potential as antimicrobial agents, which has been thought to be mainly due to the acidification of the culture medium, especially depending on the -COOH groups concentration in the material.

Polymer materials for wound dressing include hydrogels, a class of highly hydrated materials, e.g., in the form of a polymer film. In this case, antimicrobial hydrogels are especially attractive. The high water content provides a moist environment, facilitating immunological activity in the wound area, crucial during the wound-healing process. Aside from that primary function, the material imparts antimicrobial action. The nature of the biocidal activity mechanism prevents the bacteria from gaining resistance (Salomé Veiga and Schneider, 2013). Some hydrogel systems can also possess angiogenic and antioxidant abilities. In cases of deep or irregularly shaped wounds, more effective treatment may be achieved by applying injectable hydrogel (Zhong et al., 2024).

Regarding typical wound dressings, polymer films are advantageous due to their flexibility, non-invasiveness, ease of application, and ability to facilitate gas exchange. For the perfect skin treatment and repair, however, there are still years of studies ahead. The complexity and multi-parametricity of the wound repair process make it difficult to produce a dressing able to meet all the needs, making the exploration of the subject even more important (Liang et al., 2021).

In this work, citric acid and azelaic acid-based polyesters have been combined to obtain a polymer film with the idea of connecting the possible antimicrobial activity based on both the acidification of the degradable material and the intrinsic activity of the monomers. The aim was to produce and evaluate the potential of such films for topical applications targeted against microbial infections. Polyesters were synthesized, mixed, and cross-linked at different times and temperatures. Selected properties were investigated: gel content, surface hydrophilicity (water contact angle), and stability in aqueous environments (degradation tests). A modified time-kill procedure (ASTM E2315-23) was performed to test the antimicrobial properties of the films against *P. aeruginosa*, *S. aureus*, and *Candida albicans*.

## 2 Materials and methods

### 2.1 Synthesis procedure

1,5-pentanediol (Angene, 98%), 1,4-butanediol (Thermo Scientific, 98%), anhydrous citric acid (Acros Organics, ≥99.5%), azelaic acid (AmBeed, 98%) and p-toluenesulfonic acid monohydrate (PTSA; Sigma Aldrich, ≥98.5%) were used without prior preparation.

The syntheses of polyesters were carried out in a Mettler Toledo MultiMax parallel reactors system; reactors were equipped with a mechanical stirrer, temperature sensor, and DeanStark apparatus. Process parameters were dependent on the monomers used (Table 1).

**TABLE 1:** Reaction parameters.

Monomers	Reaction temperature (°C)	Reaction time
1,4-butanediol + citric acid (PTSA catalyst)	130	50 min
1,5-pentanediol + azelaic acid	150	24 h

### 2.2 NMR spectroscopy

The samples were prepared by dissolving approximately 150 mg of the sample in 1 mL of deuterated acetone. The spectra were recorded on an Agilent 400 MHz NMR spectrometer.

### 2.3 Films preparation

The pCit-pAza films were prepared by mixing the prepolymers in a mass ratio of 1:1. The mixture was weighed into a MultiMax Mettler Toledo glass reactor with a working volume of 50 mL and heated at a constant temperature of 80°C for 25 min at a constant stirring speed of 300 rpm. The still-warm prepolymer mixture was poured into a flat, rectangular Teflon form. The thermal cross-linking process was carried out in a laboratory dryer preheated to a cross-linking temperature. Cross-linking was carried out by changing two parameters: cross-linking temperature 130°C and 110°C, and cross-linking time 5, 10, 15, and 20 h for each temperature.

The pCit films were prepared similarly, except that the cross-linking temperature and time were 115°C and 20 h, respectively.

### 2.4 Gel content

Discs of 10 mm diameter were cut from the films, weighed, placed in 5 mL test tubes, and flooded with 5 mL of THF. The test tubes were placed on a Heidolph reciprocating motion shaker equipped with a thermostatic chamber. A constant temperature of 37°C and a rotation rate of 50 rpm were maintained during the test. After 24 h, the discs were removed from the solvent, dried in a laboratory dryer at 45°C for 48 h, and weighed again. Three measurements were taken for each tested material, the results were averaged, and standard deviation values were calculated.

### 2.5 Hydrophilicity

The hydrophilicity of the materials’ surface was determined by the water contact angle values. Samples were placed on the movable table equipped with the Digital Camera Industrial Digital Camera UCMOS01300KPA with Fixed Microscope Adapter FMA037. Deionized water droplets were placed onto the samples and analyzed using ToupView software. Five measurements were taken for each tested material, the results were averaged, and standard deviation values were calculated.

### 2.6 Degradation tests

Circular samples with a diameter of 1.5 cm were cut from the nonwovens, weighed, and placed in separate 5 mL Falcon tubes. 1.5 mL of freshly prepared PBS solution was added to each tube. The prepared samples were placed on a Heidolph incubation shaker equipped with a thermostatic chamber. The samples were incubated at a constant temperature of 37°C, with a reciprocating motion speed of 50 rpm. After the specified time, the discs were removed and inserted into new, clean tubes, flooded with 5 mL of deionized water, and placed back into the reciprocating shaker for 30 min at 37°C, 50 rpm. They were then placed on a Petri dish, dried in a vacuum chamber for 72 h, and weighed again. The pH value of the PBS test solutions was measured using a TFA Dostmann Check pH meter 31.3001.06. The remaining mass of the samples after degradation was calculated according to the formula:

$$remaining\;mass=\frac{m_{D}}{m_{0}}\times 100\%$$

where: *m*_D_—the sample mass after degradation, *m*_0_—the initial mass of the sample.

### 2.7 Antimicrobial efficacy testing: suspension time-kill kinetics assay

Testing of the microbicidal effect of the polycitrate and poly(citrate-*co*-azelate) films on the reference strains was performed using a modified time-kill procedure described by the American Society for Testing and Materials (ASTM)—E2315-23 standard (ASTM E2315-23, 2023). ASTM E2315-23 is used to determine the reduction of viable microorganisms over a specified time after exposure to an antimicrobial test material.

*Pseudomonas aeruginosa* ATCC 9027, *S. aureus* ATCC 6538, and *C. albicans* ATCC 10231 were used. The microorganisms were activated through double passaging: bacteria on TSA medium (Trypticase Soy Agar; BioMerieux, Craponne, France) (37°C, 24 h), and fungi on SDA medium (Sabouraud Dextrose Agar; BioMerieux, Craponne, France) (30°C, 48 h).

Overnight culture colonies of each microorganism were suspended in saline to obtain a cell density of 0.5 McFarland turbidity standard (1.5 × 10^8^ cfu/mL for bacteria and 1.5 × 10^6^ cfu/mL for yeast). The microbial cultures were then resuspended in saline to obtain a cell density of 10^4^ cfu/mL. The culture tubes were divided into three experimental groups: negative control (saline inoculated with reference microorganisms), positive control (ethanol inoculated with reference microorganisms), and polymer contact culture (saline inoculated with reference microorganisms with addition of 0.5 cm diameter discs of polymer films). Dilutions of the tested suspensions of microorganisms (10-, 100-, 1,000- and 10,000-fold) were prepared at 0, 1, 2, 3, 4, 5, 6, and 24 h of incubation (room temperature) after the polymer discs were added to the culture. Suspensions at appropriate dilutions were spread onto sterile TSA and SDA agar plates and then incubated at 37°C for 24 h for bacteria and 30°C for 48 h for *C. albicans*. The results were averaged and expressed as colony-forming units (cfu/mL). The initial and final counts in the microbial population were compared to determine the number of viable microorganism cells, the percent reduction, and the log_10_ reduction according to the formulas:

$$microbial\;reduction\;\left[\%\right]=\frac{A-B}{A}\times 100\%$$

$$\log_{10}\;reduction=\log_{10}\left(\frac{A}{B}\right)$$

where: A is the number of viable microorganisms before treatment, and B is the number of viable microorganisms after treatment.

The time-kill tests were conducted in triplicate, and the data from experiments was calculated as mean ± SD.

### 2.8 Statistical analysis

All tests were conducted in triplicate and data from experiments were calculated as mean ± SD. The standard deviation for the test of microorganism population viability does not exceed 0.5 logarithmic units.

## 3 Results and discussion

### 3.1 Polymer films characterization

**FIGURE 2:**
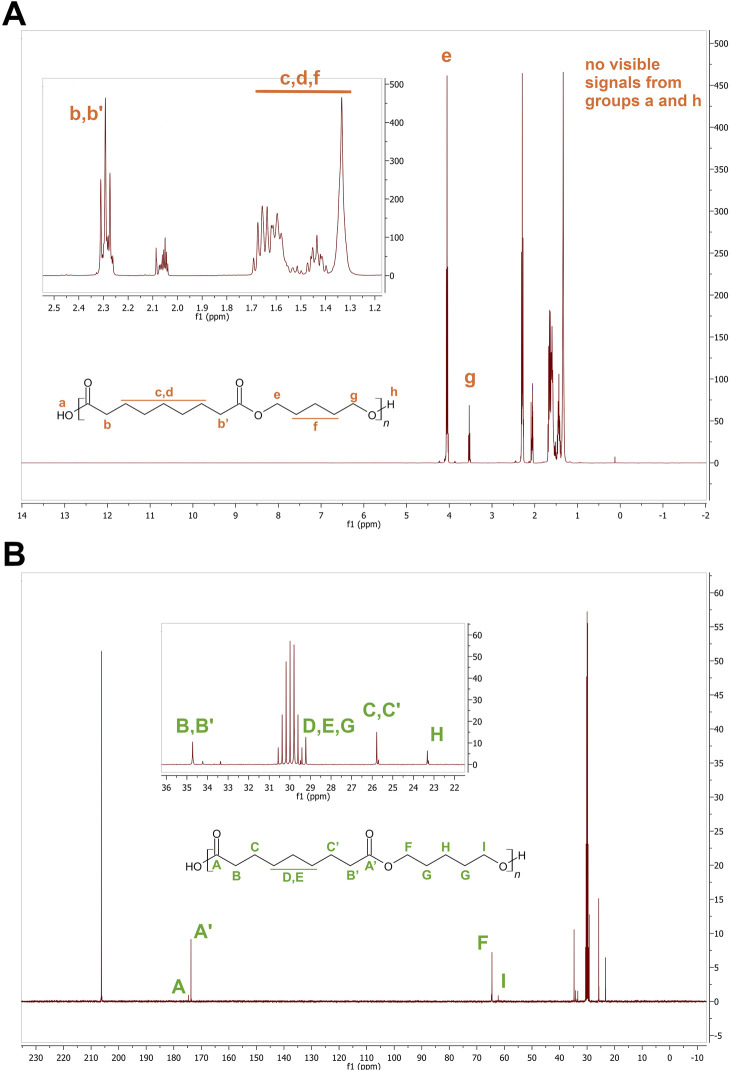
^1^H **(A)** and ^13^C **(B)** NMR spectra of poly(1,5-pentanediol azelate).

Poly(1,5-pentanediol azelate) (pAza) and poly(1,4-butanediol citrate) (pCit) have been obtained via simple polycondensation. Polyester products have been evaluated by spectral analysis. ^1^H and ^13^C NMR spectra of poly(1,5-pentanediol azelate) is provided (Figure 2). The evaluation of poly(1,4-butanediol citrate) synthesis has been reported in previous work (Bandzerewicz et al., 2023).

To obtain pCit-pAza films, polymers were mixed at a mass ratio of 1:1 and thermally cross-linked at different temperatures (110°C and 130°C) and different cross-linking times (5, 10, 15, and 20 h) to investigate the effect of conditions on the cross-linking degree of the films and the hydrophilicity of their surfaces. Films made of pCit cross-linked at 115°C for 20 h, were produced as a reference for antimicrobial testing.

**FIGURE 3:**
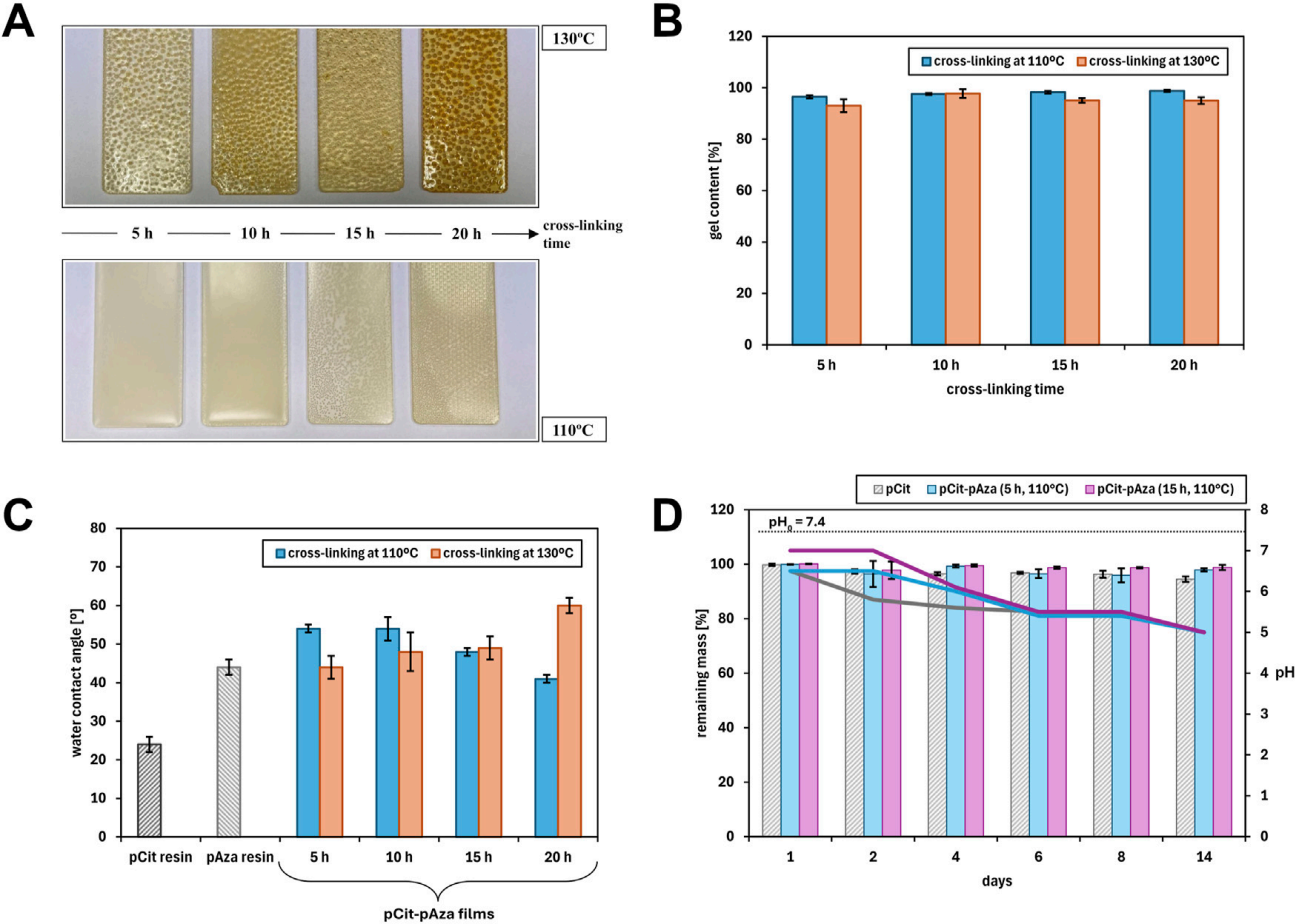
The physicochemical characterization of the pCit-pAza films obtained by applying different crosslinking times and temperatures: **(A)** color and appearance changes depending on the process patameters; **(B)** dependence of gel content on the cross-linking time and temperature; **(C)** values of water contact angle measured for pCit and pAza resins and pCit-pAza films with different cross-linking times and temperatures; **(D)** weight loss of polymer films in aqueous medium (PBS buffer) over time (bar graphs) together with changes in buffer pH (corresponding line graphs).

The pCit-pAza films obtained at 130°C were transparent and had a yellow to amber color (Figure 3A). All films contained bubbles in their structure, resulting from the formation of water as a product of the polymerization reaction during thermal cross-linking and the impediment to its evaporation from the bulk. The films obtained were relatively flexible. The longer the crosslinking time of the films was, the more brittle the films were.

The films obtained at 110°C had a milky yellow color (Figure 3A). Those cross-linked for 5 and 10 h were opaque, while films cross-linked for longer periods of 15 and 20 h were transparent. The difference in the appearance of the films may be due to the arrangement or degree of cross-linking of polyazelate in the films obtained. Polyazelates are semicrystalline and are, therefore, characterized by their opacity. In contrast, the structure of the resulting pCit-pAza copolymer is mainly amorphous: the chaotic arrangement of chains combined with their increasing length limits the possibility of crystallization. The longer the crosslinking time, the higher the conversion (esterification degree) and, thus, the amorphousness of the films (amorphous plastics are transparent).

The films obtained at both cross-linking temperatures were quite flexible. Those obtained at the lower temperature of 110°C, however, were less brittle. Their surface was also more homogeneous and contained fewer, finer bubbles in the structure. This is a result of the milder conditions - the lower cross-linking temperature allowed for slower esterification, a lower rate of water formation, and, as a result, easier removal of water from the interior and surface of the polymer films. No dependence of film adhesion on cross-linking conditions - neither cross-linking time nor temperature - was observed.

The gel content of the polymer films was determined (i.e., the content of the insoluble phase (cross-linked, gelled)) in THF, which is the solvent of both prepolymers. The results are presented in Figure 3B.

The longer the cross-linking time, the more end groups can react, so the content of the cross-linked phase should increase. For this reason, gel content values were expected to increase along with time at a given temperature. However, no such trend was observed - the gel content values for different cross-linking times are approximately the same. This may be due to two factors involved:

1. The polymers do not crosslink completely at a lower temperature and in a shorter time, but due to the high enough cross-linking density or the presence of long chains, the uncross-linked polymer does not wash out of the polymer films during the test, thus distorting the result and not giving a complete overview;
2. Cross-linking proceeds rapidly in the first stages of the process (up to the first 5 h), when the amount of free carboxyl and hydroxyl groups is maximal, and their reactivity is sufficiently high - the mobility and flexibility of the polymer chains is also the highest then. After this time, as the cross-linking time is extended, all reactions then already take place in the solid, highly cross-linked phase and no change in the properties of the polymer is apparent. The only observable changes are that of the color of the polymer films and the disappearance of the “opacity,” which are indicative of side reactions (among others, dehydration and decarboxylation) and the cross-linking of the surface layer of partially crystalline pAza, respectively.

Higher gel content values were also expected for polymer films obtained by cross-linking at higher temperatures. The gel content of films cross-linked at 130°C is slightly higher than those cross-linked at the lower temperature of 110°C.

The gel content values of all polymer films are sufficiently high that the crosslinking process can be considered successful. A particularly positive property of the polymer films was observed during the test - no loss of integrity after immersion in the test medium. In further studies, it is worthwhile to optimize the cross-linking process and to investigate the absorbability of the materials.

The water contact angle for all pCit-pAza films is less than 90° (Figure 3C), indicating the hydrophilicity of the surface of the polymer films. For pCit resin, that is, an uncross-linked material, the value is significantly lower, which is understandable, given the higher amount of hydrophilic groups per unit. After the cross-linking process, the concentration of these free groups is reduced. Apart from “the loss” of free hydrophilic groups due to their esterification, the contact angle of the film is influenced by the 50% w/w addition of the less hydrophilic pAza.

The contact angle of polymer films cross-linked at 110°C decreases with cross-linking time, while the opposite trend is observed for polymer films cross-linked at 130°C. Thus, no trend can be observed in the dependence of the contact angle on the temperature at which thermal crosslinking was carried out.

An effect of gel content on the contact angle value was expected. Because of the small differences in gel content between the polymer films, a relationship between these two parameters not be found.

The differences in the water contact angle values between the polymer films may be due to various aspects that may influence the hydrophilicity/hydrophobicity of their surface:

1. Organization of polymer chains—cross-linking time and temperature affect the arrangement of polymer chains in the film;
2. Arrangement of end groups—the arrangement of hydroxyl and carboxyl groups near the surface of the film increases the hydrophilicity of the surface;
3. Presence of by-products—during thermal cross-linking, mainly esterification occurs to produce water, but other by-products may be formed due to some possible side reactions;
4. Evaporation of water from the film—the longer the cross-linking time, the more of the generated water can be transported from the inner part of the film towards its surface; when cross-linking at a higher temperature, the evaporation of water from the surface layers should occur more easily than at a lower temperature, thereby increasing hydrophobicity with time. It is possible that at a lower temperature, the efficiency of water evaporation is lower, with more water remaining bound to the film surface, resulting in greater hydrophilicity.

### 3.2 Degradation

Short-term degradation studies of the films were performed in an aqueous medium (PBS buffer). Two pCit-pAza films were selected for this study: those cross-linked at 110°C for 5 and 15 h. The choice of films was driven by the optimization of the cross-linking conditions: a lower temperature (110°C) reduces the cost of the process. Despite the small differences in gel content values, films with different cross-linking times were chosen (5 and 15 h) to study the possible influence of the presence of incompletely cross-linked fractions of the mixture polymers not detected by the gel content tests (as discussed previously). As a reference material, a pCit-only film was tested as well. Weight loss was determined after 1, 2, 4, 6, 8, and 14 days (Figure 3D).

Over the 14 days of the degradation test, the weight loss of the films was negligible—up to a few percent. In the case of pCit films, a slight but progressive decrease in weight can be observed. This corresponds with results obtained for similar materials, with the ratio of functional groups in the prepolymer (resin) and the length of the aliphatic chains of the monomers appearing to be crucial (Bandzerewicz et al., 2024).

Some differences between the pCit-pAza and pCit films described here or in earlier studies are most likely due to the lower hydrophilicity of the polyazelate phase than that of the polycitrate phase. The longer hydrocarbon chain between the carboxyl groups in AZA makes the whole material more hydrophobic. It is also possible that the azelaic acid-derived parts of the polymer chains line up on the surface of the tested films (conclusion based on observation of an “opaque” fraction on the surface of some films), making the surface more hydrophobic. The greater hydrophobicity of the surface results in more difficult penetration of the medium into the test material hindered leaching of less cross-linked chains, and limited weight loss in the degradation test. However, as the results of pH measurements demonstrate, this does not translate into a low acidifying potential of these films.

Already after the first day of the test, a decrease in the pH value compared to the initial buffer value is visible. Over the course of the following days, phases of temporary stabilization of the pH of the medium and subsequent clear progression of acidification are evident. Changes in the first 6 days are material-dependent—the rate of acidification is increasing pCit-pAza (15 h) <pCit-pAza (5 h) <pCit. This is consistent with previous considerations regarding the influence of the hydrophilicity of the film components and the presence of less cross-linked fractions. Despite the lack of obvious hydrolytic degradation, i.e., hydrolysis of ester bonds, leaching of oligomers, and observable weight loss, the influence of the presence of free end groups in the marked decrease in pH of the medium is visible.

This may be an observation of concern in terms of the possible cytotoxicity of the materials. However, with the proposed use of this type of film being topical, it should be remembered that the pH of human skin is slightly acidic. On the other hand, some acidity in the material could support antimicrobial activity.

### 3.3 Antimicrobial activity testing

**FIGURE 4:**
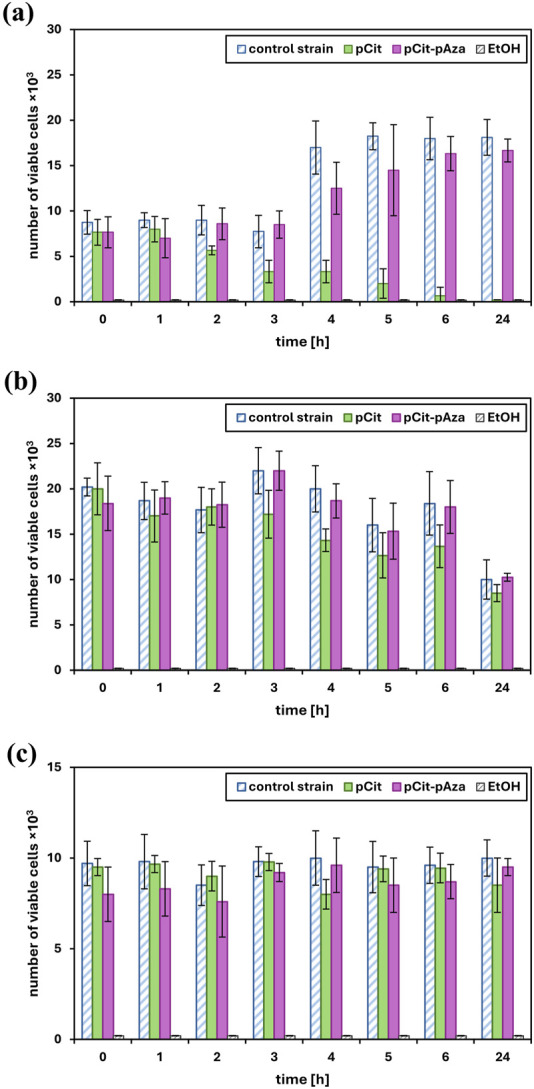
The viability of *Pseudomonas aeruginosa* ATCC 9027 **(a)**, *Staphylococcus aureus* ATCC 6538 **(b)** and *Candida* albicans ATCC 10231 **(c)**. The data is presented as the mean value ± SD of a number of viable microorganism cells in negative control (reference strains), positive control (ethanol) and polymer contact culture.

**FIGURE 5:**
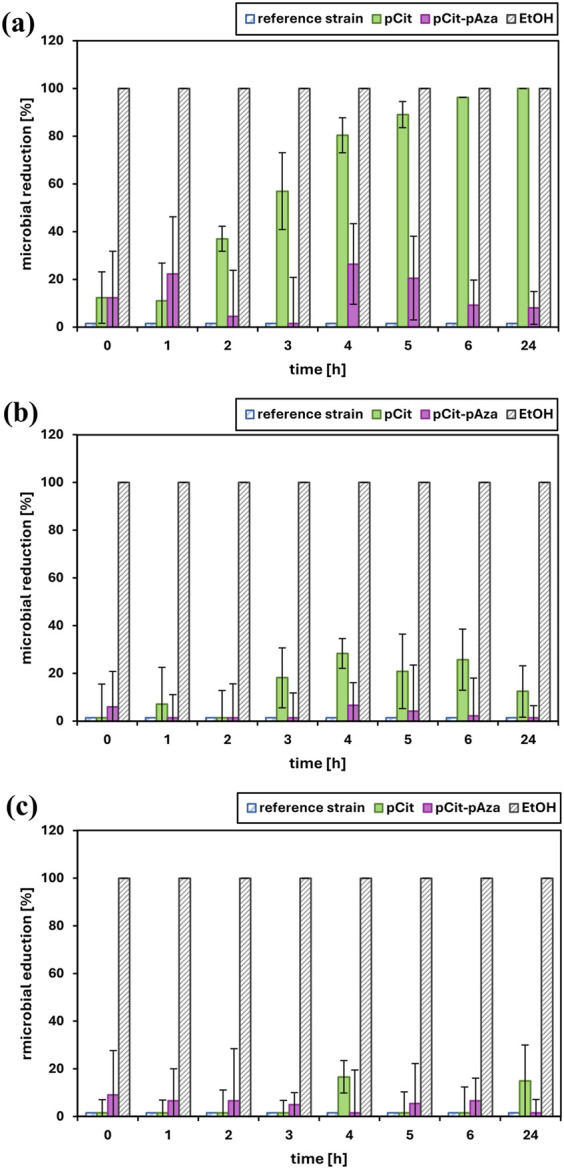
The effect of polymer films on the growth of *Pseudomonas aeruginosa* ATCC 9027 **(a)**, *Staphylococcus aureus* ATCC 6538 **(b)** and *Candida* albicans ATCC 10231 **(c)**. The data is presented as the mean value ± SD of a percentage of microbial reduction in relation to the control cultures.

**FIGURE 6:**
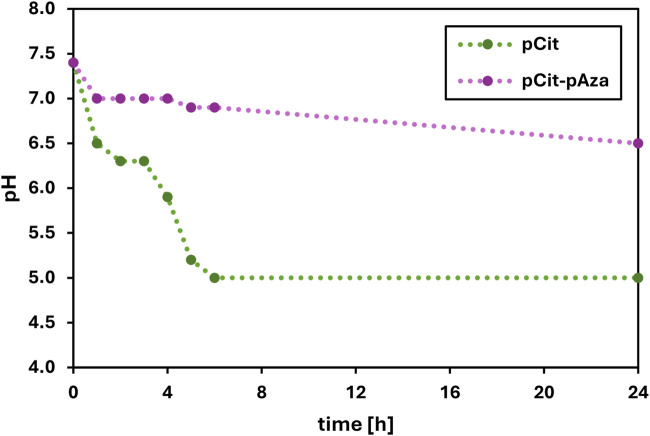
The changes in pH value of the saline after incubation with polymer film samples.

The time-kill test is a basic microbiology method for the assessment of the antimicrobial activity of a test material. For antimicrobial activity evaluation, pCit-pAza film crosslinked at 110°C for 15 h and pCit film were selected. The selection of film materials for antimicrobial test was based on the results obtained in the degradation studies, by the cost-effectiveness of the cross-linking process as well as the homogeneity of the overall film structure. The results of the viability of tested microorganisms after exposure to tested films are presented in Figure 4. The results were also calculated and expressed as a percentage of microbial reduction (Figure 5) and log_10_ reduction values (Table 2). Moreover, pH changes in the culture medium after exposure to tested materials were also measured at the respective time points (Figure 6).

**TABLE 2:** The effect of polymer films on the growth of chosen microbial strains (C. albicans, *S aureus*, and *P. aeruginosa*) calculated as a log_10_ reduction value. Ethanol (EtOH) is presented as a positive control.

Time (h)	log_10_ reduction								
	*C. albicans*			*S. aureus*			*P. aeruginosa*		
	pCit-pAza	pCit	EtOH	pCit-pAza	pCit	EtOH	pCit-pAza	pCit	EtOH
0	0.04 ± 0.02	0.00 ± 0.00	n/a	0.03 ± 0.00	0.00 ± 0.00	n/a	0.06 ± 0.05	0.06 ± 0.05	n/a
1	0.03 ± 0.01	0.00 ± 0.00		0.00 ± 0.00	0.03 ± 0.00		0.11 ± 0.05	0.05 ± 0.05	
2	0.03 ± 0.00	0.00 ± 0.00		0.00 ± 0.00	0.00 ± 0.00		0.02 ± 0.01	0.20 ± 0.03	
3	0.02 ± 0.01	0.00 ± 0.00		0.00 ± 0.00	0.09 ± 0.03		0.00 ± 0.00	0.37 ± 0.16	
4	0.00 ± 0.00	0,08 ± 0.03		0.03 ± 0.01	0.14 ± 0.04		0.13 ± 0.06	0.71 ± 0.16	
5	0.02 ± 0.00	0.00 ± 0.00		0.02 ± 0.00	0.10 ± 0.04		0.10 ± 0.05	0.96 ± 0.15	
6	0.03 ± 0.01	0.00 ± 0.00		0.01 ± 0.00	0.13 ± 0.05		0.04 ± 0.03	1.43 ± 0.01	
24	0.00 ± 0.00	0,07 ± 0.03		0.00 ± 0.00	0.06 ± 0.01		0.04 ± 0.00	n/a	

Ideally, the number of viable microbial cells in the negative control, i.e., the reference strain, should be constant throughout the test. In reality, especially with the relatively long duration of the test (24 h), it is natural that the number of viable cells will fluctuate somewhat over time depending on how well the microorganism in question is able to survive in a test medium deprived of appropriate nutrients such as saline. The viability results of the reference strains (Figure 4) show that the cells in the control were alive, which confirms that the experiment was conducted correctly (method validation).

The best results were obtained for pCit film against *P. aeruginosa*. After about 3–4 h of incubation with the polymer, there was a significant increase in microbial reduction, and after 24 h, a 100% reduction of the pathogen was achieved. The observed result is an effect of prolonged contact. The observed decrease in *P. aeruginosa* viability (increase in percentage of reduction/log_10_ reduction value) correlates very well with changes in the pH of the medium (Figure 6). Although some decrease in viability can be observed after just 1–2 h of contact, this may be due to sufficient local acidification in the medium rather than the average pH of the suspension as a whole, meaning that due to the diffusion rate of acidic oligomers/monomers/H^+^ ions, microorganisms in suspension in the closest vicinity of the film sample are killed first.

No clear biocidal effect of the pCit and pCit-pAza films was observed against *S*. *aureus*. A reduction reaching up to 30% at some time points may be indicative of an initial response of microorganisms to contact with the polymer under test, related to local changes in the pH of the sample medium (same as considered in the case of *P. aeruginosa*). It is possible that the test material did not undergo hydrolysis and leaching uniformly between the test samples.

A similar response could have been observed in the case of *C. albicans* reaction to both pCit-pAza and pCit films. At some time points, the reduction has reached up to 20%, but again, this is not a clear trend but rather isolated responses. Interestingly, in the case of this pathogen, it is for pCit that a more pronounced reduction can be observed, while pCit-pAza showed a very low but constant reduction over time. It is difficult to draw a clear conclusion from such an observation, but *C. albicans* may react to the presence of azelaic acid/azelaic oligomers in the environment while, due to the stability of the films, the concentration of these molecules was too low to observe an apparent decrease in viability.

It is quite probable that extending the incubation time would increase the antimicrobial activity of pCit-pAza, as it can be seen from Figure 6 that there is a downward pH shift between 6 and 24 h. It is difficult to predict the course of change after more than 24 h.

No shrinkage or disintegration of the films was observed during the test, so acidification of the medium is more likely to be the result of shorter chains leaching from the surface of the material or the presence of too many unreacted acid groups in polyazelate or polycitrate. As presented in Figure 6, in the case of the pCit film, the acidification of the medium is considerably faster and more pronounced, with a pH level of around five being reached after just 5 h of contact. As for the pCit-pAza film, the pH of the medium does not reach lower values than 6.5 during the entire course of the test. This is consistent with the mechanisms described earlier.

The bactericidal effect of pCit film against *P. aeruginosa* is likely due to the lowering of pH, making an environment unsuitable for the growth and multiplication of the bacteria. The acidity of the environment at the pH level of 6.3 was enough to cause about a 50% drop in the viability of bacteria. The complete reduction occurred at a pH of 5.0. In addition to a sufficiently low pH of the environment, the rate of diffusion within the test suspension is probably also an important factor.

Studies have demonstrated that *C. albicans* and *S. aureus* show sensitivity to citric acid. However, in both cases, the antimicrobial effect is attributed to the low pH (Eliuz, 2020). It has been shown that *S. aureus* is resistant to citric acid even in moderately acidic conditions (Al-Rousan et al., 2018). The results for *S. aureus* are somewhat inconsistent with the previous data concerning polymer films made of citric acid polyesters. It may be due to the differences in test procedures, whether using liquid or solid medium. To observe the biocidal effect in the case of *C. albicans*, exposure to an environmental pH ≤ 3 might be required. Similarly, the antifungal and antibacterial properties of azelaic acid against the tested pathogens are highly dependent on pH value (Charnock et al., 2004; Brasch and Christophers, 2009; Feng et al., 2024).

Both *C. albicans* and *S. aureus* have proven to be too resistant. On the other hand, the contact time might not have been long enough to cause a reduction in viability, whereas PCit-pAza film was not sufficiently acidic for any of the tested pathogens. This probably results from different degradation rates of the two polymers. The pAza fraction does not sufficiently lower the pH, while the amount of pCit is inadequate in the case of mixed material.

## 4 Conclusion

Films from a mixture of citric acid and azelaic acid polyesters were studied. It was determined that a lower cross-linking temperature (110°C) promotes less brittle and more flexible films with greater surface uniformity. No increase in gel content was observed with increasing cross-linking time. This may be due to rapid cross-linking at the beginning of the process or limited leachability of the uncross-linked polymer. The gel content value was slightly higher for cross-linking at a higher temperature. All polymer films maintained integrity after immersion in PBS buffer.

The water contact angle values indicate the surface hydrophilicity of all polymer films. The lack of unambiguous cross-linking temperature-dependent trends suggests that different aspects influence the hydrophilicity/hydrophobicity of the film surface. Factors such as the organization of the polymer chains, the arrangement of the end groups, the presence of by-products, and the efficiency of water evaporation have a significant impact on the film properties. Films cross-linked at lower temperatures show higher hydrophilicity, which may be due to lower water evaporation efficiency. Most likely, the lower hydrophilicity of the polyazelate phase limits the degradation of pCit-pAza films.

Unfortunately, pCit-pAza film has not shown antimicrobial properties against *C. albicans*, *S. aureus*, or *P. aeruginosa*, most probably due to its low acidity. However, the presented results of the antimicrobial activity of pCit film against *P. aeruginosa*, causing >90% reduction of the microorganism after 6 h of contact and 100% biocidal effect after 24 h, are a very good outlook for its future application, especially given the resistance of the pathogen to commonly used antibiotics and antiseptics applicated topically. It should be determined whether the acidity of the material is not too high and how long an exposure would be simultaneously beneficial and safe. However, a local acidic environment could positively influence the wound-healing process.

## Data Availability

The datasets presented in this study can be found in online repositories. The names of the repository/repositories and accession number(s) can be found in the article/Supplementary Material.
